# Supplementary figures and images for: LncRNA 8244-ssc-miR-320-CCR7 Regulates IFN-β during SVA Infecting PK-15 Cells
Source: Microorganisms. 2023 Mar 8;11(3):688. doi: 10.3390/microorganisms11030688 (PMC10059919; doi:10.3390/microorganisms11030688)

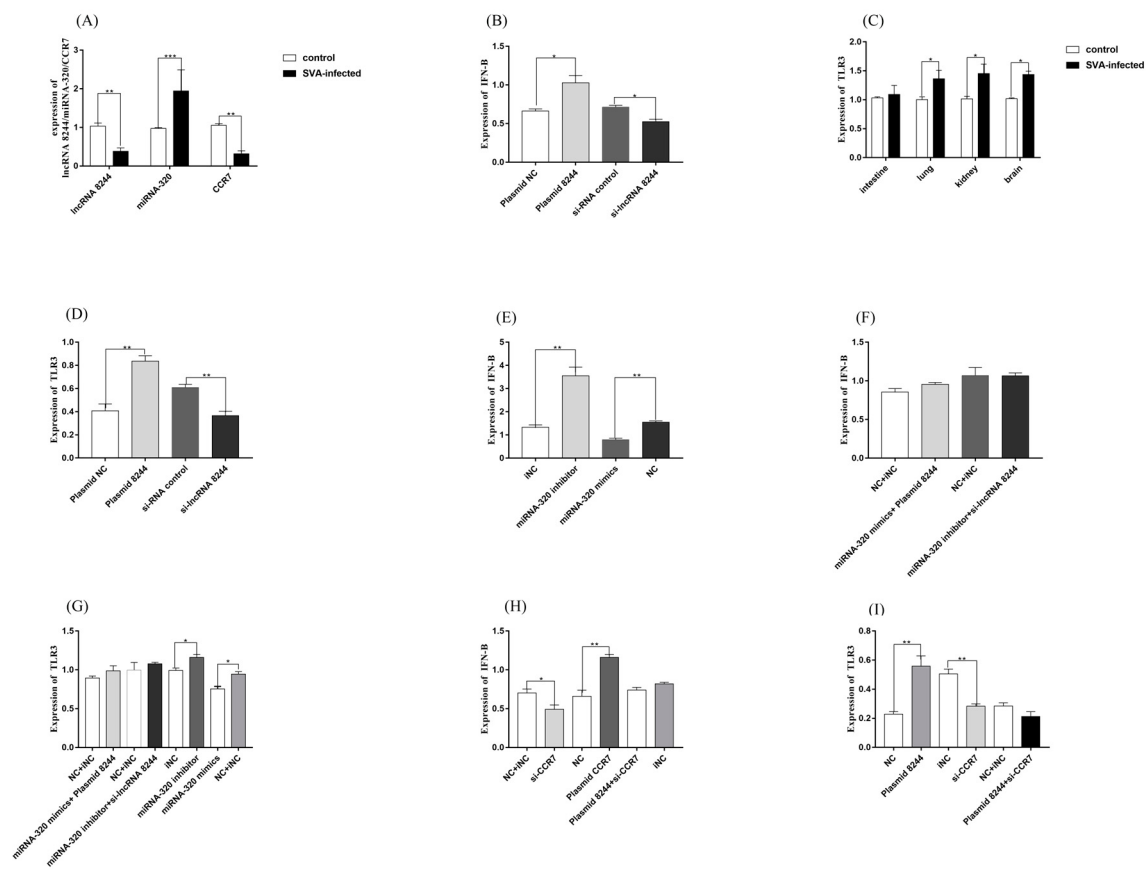

Supplement: Supplementary file 1 [file microorganisms-11-00688-s001.zip › microorganisms-2227181-supplementary.pdf]
